# HPLC analysis of CSF hypocretin-1 in type 1 and 2 narcolepsy

**DOI:** 10.1038/s41598-018-36942-8

**Published:** 2019-01-24

**Authors:** Noriaki Sakai, Mari Matsumura, Ling Lin, Emmanuel Mignot, Seiji Nishino

**Affiliations:** 1 Sleep and circadian neurobiology laboratory, School of Medicine, Stanford University, Stanford, USA; 2 Stanford center for narcolepsy, Stanford University, Stanford, USA

## Abstract

Narcolepsy is a chronic sleep disorder caused by a loss of hypocretin (hcrt) neurons in the hypothalamus. Cerebrospinal fluid (CSF) hcrt-1 measurement has been well established as a gold standard of narcolepsy diagnosis, although some portions of narcoleptic patients show normal hcrt-1 levels. We aimed to examine peptide degradation of hcrt-1 and its abnormality in the CSF of patients by using high performance liquid chromatography (HPLC) followed by radioimmunoassay (RIA). CSF was collected from healthy controls, narcoleptic patients of type 1 with hcrt-1 deficiency, type 1 with normal hcrt-1 level, and type 2 with normal hcrt-1 level. We found that the majority of hcrt-1 immunoreactivity in extracted CSF was derived from unauthentic hcrt-1 peaks, which are predicted to be inactive metabolites, and the intact hcrt-1 peptide was less than 10% of the gross amount, suggesting that the regular RIA for CSF hcrt-1 measures largely reflect the unauthentic hcrt-1-related metabolites rather than the intact one. As expected, all hcrt-1-related peaks were abolished in type 1 with hcrt-1 deficiency. Importantly, we also found that the sum of the authentic hcrt-1 peptide (peaks 3 and 4) significantly decreased in non-deficient type 1 and tended to decrease in type 2 narcoleptic patients although the levels with the regular RIA in non-extracted CSF was equivalent to healthy controls. Immunoreactivity with unauthentic hcrt-1 metabolites may masks the possible decline in authentic hcrt-1 level caused by the partial loss of hcrt neurons. Our findings may provide new insights into the degradation of the hcrt-1 peptide and the pathophysiology of narcolepsy.

## Introduction

Narcolepsy is a chronic sleep disorder characterized by excessive daytime sleepiness, cataplexy, sleep paralysis, and hypnagogic hallucinations. The hypocretin (hcrt)/orexin system plays pivotal roles in the etiology of narcolepsy^1^. Since reduced number of hcrt cells are observed in narcolepsy with cataplexy patients and a mutation in hcrt-related genes is extremely rare in human cases^2,3^, CSF hcrt-1 measurement has been well established as a gold standard of diagnosis of narcolepsy by the ICSD-2^4,5^. However, it has been reported that 10% of narcolepsy with cataplexy and 80–90% of narcolepsy without cataplexy cases have normal CSF hcrt-1 levels^6^. In addition, normal or moderate hcrt-1 level is one of the current diagnostic criteria for type 2 narcolepsy. However, mechanisms underlying normal hcrt-1 level in narcolepsy are largely unknown and studies focusing on hcrt-1 non-deficient type 1 patients are very limited. Therefore, its clinical features are heterogeneous and the discrepancy between normal hcrt-1 level and clinical symptoms could make the differential diagnosis complex.

Hcrt-1 and -2 are derived from a single precursor polypeptide, prepro-hypocretin, through proteolytic cleavage. Hcrt-1 is a 33-amino acid peptide with a N-terminal pyroglutamyl residue and C-terminal amidation. Hcrt-1 possesses two intrachain disulfide bonds that consist of four cysteine residues (Cys6-Cys12 and Cys7-Cys14) in its N-terminal, and these bonds make a rigid turn conformation^7,8^. The peptide sequence and structure are highly conserved across mammalian species. On the other hand, hcrt-2 is a 28-amino acid with C-terminal amidation. Unlike hcrt-1, its N-terminal half does not include disulfide bonds and thus forms a linear peptide, although its C-terminal half is very similar to that of hcrt-1. Both of the two peptides are exclusively produced in the lateral hypothalamic area. Hcrt neurons widely project to multiple areas throughout the brain except the cerebellum^9–11^. Among them, dense projection is observed in the serotonergic raphe nuclei, histaminergic tuberomammillary nucleus, and noradrenergic locus coeruleus where hcrt-1 receptor and/or hcrt-2 receptor are expressed, suggesting that hcrts and their receptors are likely to regulate not only the stability of wake and sleep states but more broadly of brain functions such as feeding, emotion, and reward.

Studies focusing on narcolepsy without cataplexy have so far been limited due to a more heterogeneous etiology, such as less frequent HLA DQB1*06:02 positivity^12–14^, low rates (0–40%) of hcrt-1 deficiency^6,12,15–20^, and inconsistency in multiple sleep latency test results^21^. In addition, nonspecific symptoms further make the diagnosis of narcolepsy without cataplexy challenging^22^. A postmortem study reported that a narcolepsy without cataplexy patient showed a partial loss of hcrt cells in the posterior hypothalamus, accompanied by prominent gliosis, but not in the anterior hypothalamus^23^. However, this observation was reported in a single case, and partial hcrt cell loss needs to be further confirmed in more patients. Additionally, evidence linking a focal loss and an absence of cataplexy remains unclear.

To date, little is known about degradation of the hcrt-1 peptide in the CSF. In addition, the regular RIA provides the total value of peptides that are immunoreactive to an anti-hcrt-1 antibody, but it has not been validated if the regular RIA reflects the intact hcrt-1. To address the discrepancy in the CSF hcrt-1 level and nosological aspects of type 1 and 2 narcolepsy, it is important to characterize the degradation of hcrt-1 peptide in type 1 and 2 narcolepsy patients. In this study, we analyzed the HPLC elution pattern and quantified antibody immunoreactive fractions in the CSF.

## Methods

### Ethics statement

All methods were performed in accordance with the relevant guidelines and regulations. Informed consent in accordance with Stanford guidelines was obtained from all subjects. The research protocol was approved by the Stanford Institutional Review Board on Medical Human Subjects.

### Subjects

CSF samples from 23 narcoleptics and 10 healthy control subjects were selected from the database at the Stanford University Center for Narcolepsy as previously described^6,24,25^. All narcoleptics had been diagnosed based on the clinical symptoms and CSF hcrt-1 level according to ICSD-2 and were regrouped in this study according to ICSD-3 as follows: type 1, narcolepsy-cataplexy with hcrt-1 deficiency (n = 6); non-deficient type 1, narcolepsy-cataplexy with normal levels of hcrt-1 (n = 12); non-deficient type 2, narcolepsy without cataplexy with normal levels of hcrt-1 (n = 5). All control subjects had no clinical sleep abnormalities. CSF samples have been collected by lumber puncture between the years of 1995 and 1997 (between 9 am and 7 pm) from patients and healthy subjects for narcolepsy diagnosis and research purposes, and have been kept frozen at −80 °C. Demographics of samples used are summarized in Table 1.

**Table 1:** Demographics of CSF samples.

	Control (N = 10)	Type 1		Type 2
		Deficiency (N = 6)	Normal (N = 12)	Normal (N = 5)
DQB1*06:02 positive	3/8	5/6	3/12	1/4
Gender (Male, Female)	7, 3	4, 2	5, 7	2, 3
Age (years)	44.9 ± 6.0	34.5 ± 3.8	35.8 ± 2.8	31.8 ± 4.8
MSLT Latency (min)	N/A	3.2 ± 0.8	4.8 ± 0.9	3.4 ± 0.7
Number of SOREMP	N/A	3.0 ± 0.3	2.1 ± 0.6	2.4 ± 0.2
Medication (number)	no medication (10)	Provigil (1)	Provigil (5)	Provigil (2)
		Ritalin (1)	Ritalin (3)	Amphetamine (1)
		Prozac (1)	Prozac (1)	Prozac (1)
		GHB (1)	GHB (1)	Celexa (1)
		Amphetamine (1)	Dexedrine (1)	Dexedrine (1)
		no medication (2)	Caffeine (2)	no medication (2)
			Desipramine (1)	
			Klonopin (1)	
			Paxil (1)	
			Desoxyn (1)	
			Sinemet (1)	
			Temazepam (1)	
			no medication (1)	
			N/A (1)	

MSLT; multiple sleep latency test, SOREMP; sleep onset REM sleep period.

### HPLC separation

All CSF samples have gone through freeze-thaw cycles of less than 3 times before the experiment. CSF samples were centrifuged at 10,000 g for 10 minutes and the supernatant was used for analysis. One ml of CSF samples from patients of type 1, non-deficient type 1, non-deficient type 2, and healthy control subjects were separated by HPLC (Model 526 HPLC pump, Alltech) at a flow rate of 1 ml/min and linear gradient of 10–60% acetonitrile/0.1% trifluroacetic acid over 40 minutes. Samples were separated by μBondapak C18 column 3.9 × 300 mm, 10 μM 125 A, (Waters Corporation, Milford, MA). Fractions were collected every minute over 40 minutes and were dried up using a vacuum centrifuge system (SpeedVac, Savant). Samples were stored at −20 °C until use. In order to identify the authentic hcrt-1 peptide, ^125^I labeled Orexin A isotope (T-003-30, Phoenix Pharmaceuticals, Burlingame, CA) was separated using the same protocol.

### Radioimmunoassay

Hcrt-1 concentration in the CSF with and without extraction was measured using an in-house hcrt-1 antibody and ^125^I labeled Orexin A isotope according to the manufacture’s instruction. The in-house polyclonal anti-hcrt-1 antibody was raised in rabbits by injecting a full-length hcrt-1 peptide. The IC90 of the RIA was 0.5 pg/ml, which about 20 times more sensitive than the commercially available antibody by Phoenix. A high sensitive assay is more suitable for the purpose of the study and we therefore used our in-house antibody for the RIA. The antibody did not cross-react with hcrt-2 and other related peptides such as the following: growth hormone releasing factor, glucagon, vasoactive intestine peptide, and secretin that belong to the secretin family peptides, bombesin, gastrin releasing peptide, and neuromedin C and B that belong to the bombesin family peptides (unpublished data). The intra- and inter-assay variations were determined by measuring 5 triplicated samples on 3 different days (a total of 45 assays). Intra-assay coefficient of variation was between 3.5 and 6.1%, while inter-assay coefficient of variation was between 2.5 and 6.4%. We also confirmed that the antibody could successfully detect hcrt-1 neurons in rats, zebra fish, dogs, and humans (unpublished data). For the measurement of hcrt-1 immunoreactive peaks in the CSF, fractioned samples were resuspended in 250 µl of deionized water, and 100 µl was used in duplicates for the assay. Radioactivity of each fraction was quantified with a gamma counter (Cobra II, Packard). All values are expressed as mean ± SEM. All procedures including human CSF and radioactive material handling were carried out in accordance with guidelines of Stanford Environmental Health and Safety.

### Statistical analysis

The mean values of hcrt-1 of the 1-minute fractions were plotted as an elution pattern. The area under curve of each peak was calculated and compared among groups using a Kruskal-Wallis test with post-hoc testing using Steel test (JMP).

## Results

### Hcrt-1 immunoreactivity is mainly not the intact hcrt-1 peptide

**Figure 1:**
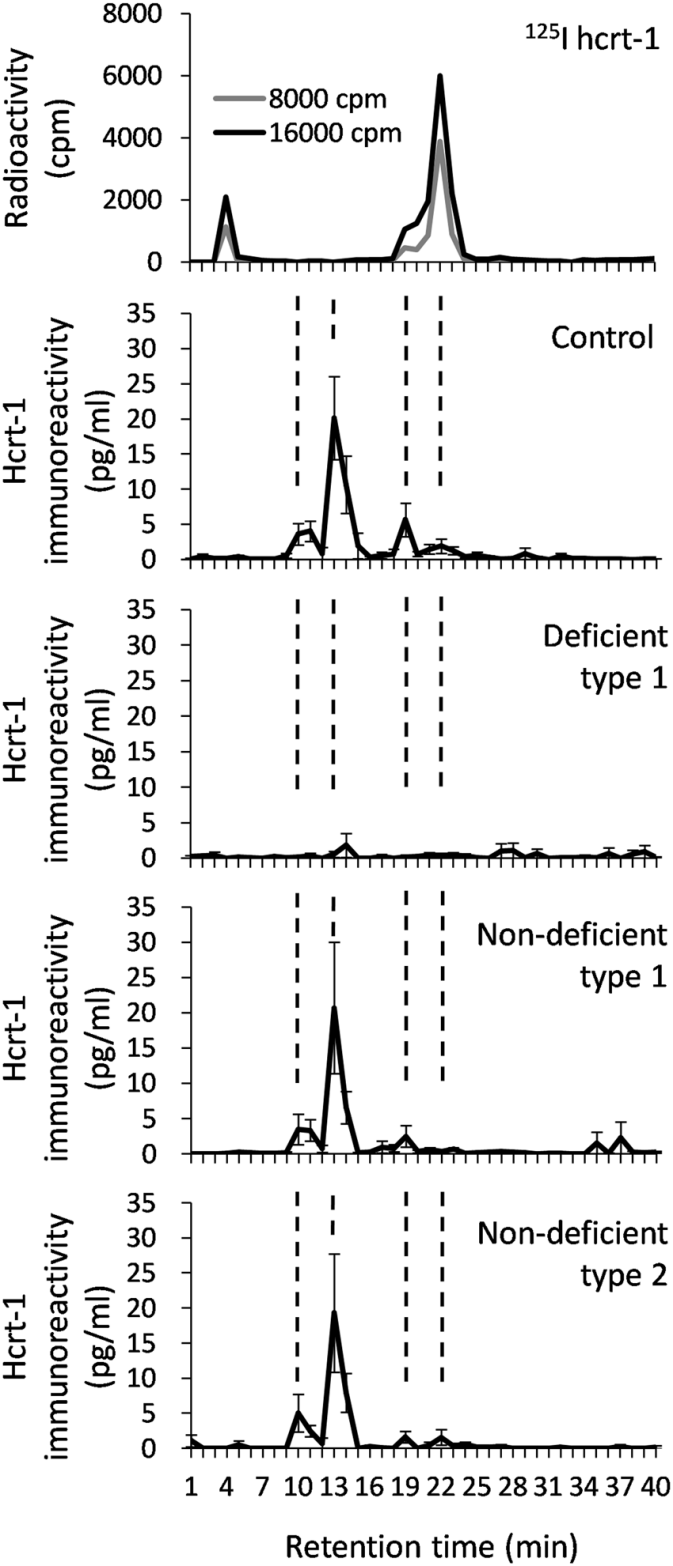
Elution patterns of ^125^I labeled hcrt-1 and CSF samples. The mean value of RIA in each 1-minute fraction was plotted. Dashed lines show each distinguishable peak at 9–12, 12–15, 18–20, and 20–23 minutes. Error bar represents SEM.

In order to determine the retention time of the authentic hcrt-1 peak, ^125^I-labeled hcrt-1 was fractionalized and measured by RIA. Of the 3 peaks at 3–5, 18–20, and 20–23 minutes, the small peak at 18–20 minutes and the following major peak at 20–23 minutes likely correspond to the authentic hcrt-1. A synthetic hcrt-1 peptide (160 pg of the standard peptide included in a RIA kit) was also separated using HPLC, and a major peak was observed at 18–23 minutes (small and major authentic peaks were combined, data not shown). Four distinguishable peaks (peak 1; 9–12, peak 2; 12–15, peak 3; 18–20, and peak 4; 20–23 minutes) were observed in the CSF from healthy subjects, non-deficient type 1, and non-deficient type 2 (Fig. 1). The same elusion pattern was observed in freshly collected or more than 3 times-freeze-thawed CSF (data not shown). The majority of hcrt-1 immunoreactivity was derived from an unknown peak 2. Although non-deficient type 1 and type 2 narcolepsies showed a tendency to decrease in peaks 3 and 4 compared with control, they did not reach statistical significance. However, the difference became more evident when these peaks were combined. Non-deficient type 1 reached statistical significance (Z = −2.47, *p* = 0.03). On the other hand, non-deficient type 2 failed to reach the significance (Z = −1.28, *p* = 0.44) because 2 out of 5 samples showed equivalent values to healthy control. Interestingly, the hcrt-1 concentration in non-extracted CSF among these groups was equivalent to the healthy control group (Table 2). As expected, hcrt-1 deficient type 1 had no overt peaks (peak 1; Z = −3.11, *p* = 0.005, peak 2; Z = −2.99, *p* = 0.008, peak 3; Z = −2.99, *p* = 0.008, sum of peaks 3 and 4; Z = −2.55, *p* = 0.029, total peaks; Z = −3.20, *p* = 0.004).

**Table 2:** Comparison of the hcrt-1 concentration immunoreactive to the antibody in the CSF with and without extraction.

	Control (pg/ml)	Type 1		Type 2	*P-*value
		Deficiency (pg/ml)	Normal (pg/ml)	Normal (pg/ml)	
***Non-extracted CSF***					
Hcrt-1	250.0 ± 15.8	<40.0***	282.0 ± 6.7	248.0 ± 4.0	0.000
***Extracted CSF with HPLC***					
Peak 1 (9–12 min)	8.4 ± 2.2	0.6 ± 0.4**	7.3 ± 3.6	8.0 ± 3.3	0.007
Peak 2 (12–15 min)	32.2 ± 8.4	2.4 ± 2.1**	27.8 ± 9.7	27.7 ± 8.2	0.005
Peak 3 (18–20 min)	6.4 ± 2.4	0.4 ± 0.2**	3.1 ± 1.5	1.7 ± 0.8	0.015
Peak 4 (20–23 min)	4.4 ± 1.3	1.1 ± 0.9	1.5 ± 0.5	2.2 ± 1.4	0.160
Sum of Peak 3 & 4	10.9 ± 3.3	1.5 ± 0.9*	4.6 ± 1.9*	3.9 ± 2.2	0.019
Total peaks	51.5 ± 13.6	4.5 ± 2.6*	39.6 ± 14.3	39.6 ± 11.8	0.028

Kruskal-Wallis and a post-hoc testing using Steel test were applied. *p < 0.05; **p < 0.01; ***p < 0.001.

Since RIA using a polyclonal antibody from Phoenix is widely used for clinical diagnosis test for narcolepsy, we also characterized Phoenix and our in-house antibodies: hcrt-1 levels in extracted CSF from a healthy subject and immunohistochemistry in a mouse brain were compared (Supplement Fig. 1). Peaks at 10–11 and 13–14 minutes were successfully detected by both antibodies. Since in-house antibody has much higher sensitivity to hcrt-1, the small authentic peak at 19 minute was detected by in-house antibody but not Phoenix one. The major authentic peak was undetectable in this CSF sample, suggesting that intact hcrt-1 peptide is extremely low even in healthy subjects. In immunohistochemistry, hcrt-producing neurons were successfully stained by both antibodies. In light of these results, we assume that our in-house antibody shares similar characteristics with the Phoenix antibody.

## Discussion

The major aim of the study was to examine if abnormal degradation of hcrt-1 peptide occurred in type 1 and 2 narcolepsy patients. Hcrt-1 and hcrt-2 are produced from a common precursor prepro-hypocretin in a limited number of neurons in the lateral hypothalamic area. Compared with anatomical and physiological studies of the hcrt system, little is known on how hcrt can be degraded. The abnormal degradation of peptides/proteins is involved in the pathology of neurological disorders such as Alzheimer’s disease, Parkinson’s disease, and prion disease^26^. However, peptide degradation of hcrt-1 and its abnormality in narcoleptic patients still remains unknown.

To this end, we determined fractions separated by HPLC that are immunoreactive to anti-hcrt-1 antibody. Surprisingly, in healthy controls, the majority of immunoreactivity was derived from unknown peaks, and the intact hcrt-1 peptide was less than 10% of the sum of 4 peaks, suggesting that the regular RIA measure of non-extracted CSF largely reflects the quantity of unauthentic peaks more than that of intact hcrt-1. This is likely true in case of RIA using the Phoenix antibody that is most commonly used for the diagnostic test (see Supplement Fig. 1). Since all peaks were detected even in a fresh CSF collected in a same day that was kept on ice until separated with HPLC, and repeated freeze-thaw cycles did not affect the peaks, the unknown peaks are not due to artificial decays during the handling or storage of the CSF samples. Most importantly, all peaks decreased significantly to nearly undetectable levels in hcrt-deficient type 1, suggesting that unknown peaks are metabolites of authentic hcrt-1. A combined study of the peptidome and proteome of human CSF identified two cysteine-containing peptide variants (residues 1–14 and 1–16) derived from the N-terminal part of hcrt-1^27^. Many truncation studies have reported that the lipophilic C-terminal part of hcrt-1, which is a highly conserved sequence between hcrts, is required for receptor interaction and functional potency^28–32^. Thus, the two peptide variants are most likely to be biologically inactive and resistant to degradation. Although we were not able to identify the unknown peaks in the current study, we assume that either or both of the peaks 1 and 2 might be 2 peptide variants of the N-terminal. If this assumption is correct, the hcrt-1 peptide would be mostly degraded in the CSF or degraded peptides be secreted into the CSF, implying that the lumbar CSF would largely reflect the amount of inactive metabolites of hcrt-1. The controversial issue surrounding RIA is also likely due to the antibodies used for the assay since the overall values of immunoreactive hcrt-1 between hcrt-deficient and non-deficient subjects and their separations were significantly different depending on the antibodies^33,34^. Recently, an innovative quantitative method of quantifying hcrt-1 was developed by using liquid chromatography combined with mass spectrometry in the multiple reaction monitoring mode^35^. Despite its high specificity against hcrt-1, the values had a wider distribution and were 2.5 times lower than the regular RIA measures. These differences in values between the regular RIA and the mass spectrometry technique may also be explained by our finding that the intact hcrt-1 peptide occupies a small portion of total RIA values. Of note, our finding does not compromise the diagnostic values of the regular CSF measure because all cases of type 1 with hcrt deficiency showed no discrepancy in RIA values between non-extracted and extracted CSF.

It has been reported that narcolepsy without cataplexy (type 2) patients have a partial loss of hcrt cells, with maximal cell loss in the posterior hypothalamus^23^. In this study, both non-deficient type 1 and type 2 showed all immunoreactive peaks. The amount of the major unknown peaks 1 and 2 in both groups was equivalent to that in healthy controls. Considering the facts that the peak 3 was observed in ^125^I-labeled hcrt-1 fraction and hcrt-1 possesses amphiphilic structures, the peak 3 may be biologically functional and form self-aggregate or dimer of hcrt-1 peptide^7^. These data imply that hcrt cells would survive to some extent. In addition, the sum of authentic peaks revealed that hcrt neurotransmission may be partially but more severely compromised in non-deficient type 1 than non-deficient type 2, presumably resulting in the pathophysiological difference such as occurrence of cataplexy. Thus, our finding would in part support the observation in the postmortem brain of narcolepsy without cataplexy. Additionally, normal CSF hcrt-1 level in non-deficient type 1 and type 2 is possibly explained by non-authentic hcrt-1-related peaks.

Since the RIA measure gives relative value and is methodologically not superior in quantification of small differences, it would be of interest to measure the hcrt-1 level in non-deficient type 1 and type 2 narcolepsy by more specific quantitative methods. It also raises the possibility that hcrt-1 peptide decay might be accelerated in non-deficient cases, yet not much is known about the degradation process of hcrts. Some factors that may influence the degradation deserve consideration. In this study, we used CSF samples that were stored at −80 °C for a long period. Advanced decay during storage could be ruled out because freshly collected CSF also showed a similar peak pattern and the authentic hcrt-1 level was already low. It is difficult to speculate on the effect of medication on hcrt degradation from only 1 or 2 non-medicated patients in each group. It has been reported that CSF hcrt-1 level shows seasonal fluctuation in humans and rodents and with a clear diurnal rhythm^36,37^. We also cannot exclude the possibility of circadian modulation in degradation because little information is available concerning degradation process of hcrts. Further studies are needed to address these issues and to determine if the HPLC peak pattern we reported here is generally observed in narcoleptic patients with normal hcrt-1 level.

## Supplementary information

Supplementary information
