# Supplementary material for: HPLC analysis of CSF hypocretin-1 in type 1 and 2 narcolepsy
Source: Sci Rep. 2019 Jan 24;9:477. doi: 10.1038/s41598-018-36942-8 (PMC6346108; doi:10.1038/s41598-018-36942-8)
Supplement: Supplementary file 1 — Supplementary information [file 41598_2018_36942_MOESM1_ESM.pdf]

Supplementary information

Title

HPLC analysis of CSF hypocretin-1 in type 1 and 2 narcolepsy

Noriaki Sakai[1¶]; Mari Matsumura[1¶]; Ling Lin[2]; Emmanuel Mignot[2]; Seiji Nishino[1*]

1, Sleep and circadian neurobiology laboratory, School of Medicine, Stanford University

2, Stanford center for narcolepsy, Stanford University

¶These authors equally contributed.

Supplementary Material and Method

Immunohistochemistry

A C57BL/6J (#664, The Jackson Laboratory) was euthanized and the brain was dissected in 3 mm thickness including the hypothalamus. After fixation of 4% paraformaldehyde solution, the specimen was immersed in 30% sucrose in PBS overnight followed by cryosection. Sections were subjected to DAB staining. The in-house antibody was diluted by 20 times with RIA buffer, while Phoenix antibody was prepared as recommended in the manufacturer's instruction and used for histology with no further dilution. Photographs were captured using Moticam 5+.

Supplemental fig. 1

a

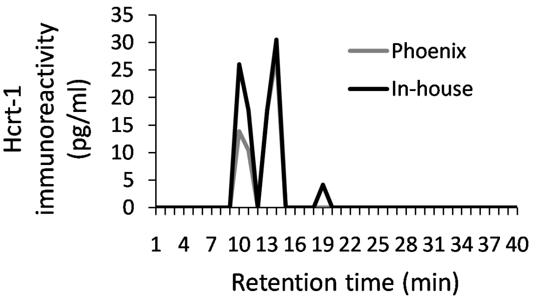

b

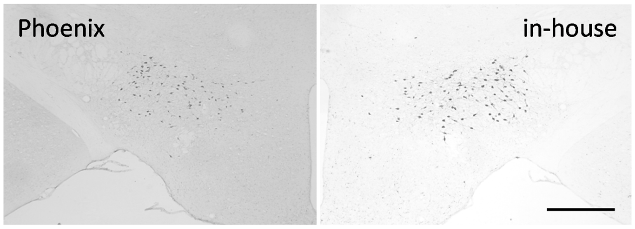

Supplementary Figure legend

Supplementary figure 1 - Comparison of Phoenix and in-house antibodies

a) A CSF sample from a healthy subject was used for the comparison. One-minute-fractions were resuspended in 250 µl of water and split into half for RIA using Phoenix and in-house antibodies. b) Immunohistochemistry was performed in a C57BL/6J mouse brain using Phoenix and in-house antibodies. Scale bar, 500 µm.
